# History of autoimmune conditions and lymphoma prognosis

**DOI:** 10.1038/s41408-018-0105-4

**Published:** 2018-08-01

**Authors:** Geffen Kleinstern, Matthew J. Maurer, Mark Liebow, Thomas M. Habermann, Jean L. Koff, Cristine Allmer, Thomas E. Witzig, Grzegorz S. Nowakowski, Ivana N. Micallef, Patrick B. Johnston, David J. Inwards, Carrie A. Thompson, Andrew L. Feldman, Brian K. Link, Christopher Flowers, Susan L. Slager, James R. Cerhan

**Affiliations:** 1 Department of Health Sciences Research, Mayo Clinic, Rochester, MN USA; 2 Division of General Internal Medicine, Mayo Clinic, Rochester, MN USA; 3 Division of Hematology, Mayo Clinic, Rochester, MN USA; 4 Division of Bone Marrow and Stem Cell Transplantation, Winship Cancer Institute of Emory University, Atlanta, GA USA; 5 Department of Laboratory Medicine and Pathology, Mayo Clinic, Rochester, MN USA; 6 University of Iowa Hospitals and Clinics, Iowa City, IA USA; 7 Winship Cancer Institute of Emory University, Atlanta, GA USA

## Abstract

Autoimmune conditions are strong risk factors for developing lymphoma, but their role in lymphoma prognosis is less clear. In a prospective cohort study, we evaluated self-reported history of eight autoimmune conditions with outcomes in 736 diffuse large B-cell, 703 follicular, 302 marginal zone (MZL), 193 mantle cell (MCL), 297 Hodgkin lymphoma (HL), and 186 T-cell lymphomas. We calculated event-free survival (EFS) and overall survival (OS), and estimated hazard ratios (HRs) and 95% confidence intervals (CIs), adjusting for sex, prognostic score, and treatment. History of any of the eight autoimmune conditions ranged from 7.4% in HL to 18.2% in MZL, and was not associated with EFS or OS for any lymphoma subtype. However, there was a positive association of autoimmune conditions primarily mediated by B-cell responses with inferior EFS in MCL (HR = 2.23, CI: 1.15–4.34) and HL (HR = 2.63, CI: 1.04–6.63), which was largely driven by rheumatoid arthritis. Autoimmune conditions primarily mediated by T-cell responses were not found to be associated with EFS or OS in any lymphoma subtype, although there were few events for this exposure. Our results indicate that distinguishing autoimmune conditions primarily mediated by B-cell/T-cell responses may yield insight regarding the impact of this comorbid disease, affecting ~10% of lymphoma patients, on survival.

## Introduction

Lymphomas are a heterogeneous group of malignancies that account for ~3–4% of cancers worldwide^1^. Non-Hodgkin lymphoma (NHL) and Hodgkin lymphoma (HL) are histologically and genetically diverse, and may originate from either B- or T-lymphocytes^2,3^. Autoimmune conditions, which affect ~3% of the general population^4^, are an established risk factor for lymphoma, conferring ~2- to 37-fold increased risk^5–12^. Although there are over 80 autoimmune conditions, they can be broadly classified as primarily mediated by B-cell responses or T-cell responses, acknowledging some overlap^13–16^. Representative B-cell-mediated autoimmune diseases include rheumatoid arthritis (RA) and systemic lupus erythematosus (SLE), and representative T-cell-mediated diseases include celiac disease and ulcerative colitis.

In a large pooled analysis from the International Lymphoma Epidemiology Consortium (InterLymph) of 17,471 NHL cases and 23,096 controls, autoimmune conditions classified as primarily mediated by B-cell responses were associated with an increased risk of lymphoma, particularly diffuse large B-cell lymphoma (DLBCL) and marginal zone lymphoma (MZL), whereas autoimmune conditions classified as primarily mediated by T-cell responses were only associated with risk of T-cell lymphoma (TCL)^10–12,17^. In contrast to lymphoma etiology, relatively few studies have evaluated the relationships between history of autoimmune conditions with lymphoma prognosis^18–24^, which may have implications for clinical management. We evaluated lymphoma subtype-specific outcomes by autoimmune history overall, as well as classified as autoimmune conditions primarily mediated by B-cell responses or T-cell responses in a prospective cohort study with detailed clinical, treatment, and outcome data.

## Methods

We used Mayo Clinic cases enrolled in the University of Iowa/Mayo Clinic SPORE Molecular Epidemiology Resource, a prospective cohort study that has been previously described^25^. Briefly, consecutive patients with lymphoma were prospectively approached within 9 months of diagnosis for enrollment. Pathology was centrally reviewed and classified according to the World Health Organization^26^. Clinical and treatment data were abstracted using standard protocols, and participants were contacted every 6 months for the first 3 years, then annually to ascertain disease recurrence or progression, new treatments, transformation, and new cancer diagnoses. All events were validated against medical records.

All participants provided written informed consent and the cohort protocol was approved by the institutional review boards at the Mayo Clinic.

Participants enrolled at Mayo Clinic from 2002–2015 with self-reported risk factor data on 8 autoimmune diseases were eligible for the current analysis, which included 736 DLBCL, 703 follicular lymphoma (FL), 302 MZL, 193 mantle cell lymphoma (MCL), 297 HL, and 186 TCL patients. Autoimmune conditions were categorized as either primarily mediated by B-cell responses [RA, Sjögren syndrome (SS), SLE, and Hashimoto thyroiditis] or T-cell responses [celiac disease, Crohn’s, ulcerative colitis, and polymyositis/dermatomyositis] according to the InterLymph classification^27^.

The *χ*^2^-test was used to calculate associations between overall autoimmune conditions and lymphoma patients’ clinical characteristics such as sex, age, Eastern Cooperative Oncology Group (ECOG) performance status, and prognostic index. We defined event-free survival (EFS) as time from diagnosis to progression/relapse, re-treatment, or death, and overall survival (OS) as time from diagnosis to death due to any cause. We used Cox proportional hazards regression analysis to estimate hazard ratios (HRs) and 95% confidence intervals (CIs) to test the association between autoimmune conditions and EFS and OS, adjusting for sex and the following subtype-specific variables: International Prognostic Index (IPI)^28^ for DLBCL, MZL, and TCL; Mantle Cell IPI (MIPI)^29^ for MCL; International Prognostic Score (IPS)^30^ for HL; FLIPI^31^, FL grade III, and treatment (observation, rituximab monotherapy, immunochemotherapy, and other therapy**)** for FL; and treatment [immunochemotherapy (anthracyline based) vs. other] for DLBCL. In additional modeling, we also adjusted for presence of any of seven selected comorbidities (other cancer diagnosis within 3 years of lymphoma diagnosis [excluding non-melanoma skin cancer], coronary heart disease, congestive heart failure, diabetes, hip fracture, hepatitis, and elevated creatinine) and smoking status (never, former, and current). Finally, to estimate the association of autoimmune disease across all six lymphoma subtypes for EFS and OS, we conducted a meta-analysis using the fixed-effects method. We calculated Cochran’s *Q*-statistic to test for heterogeneity across lymphoma subtypes and the *I*^2^ statistic to quantify the proportion of the total variation due to heterogeneity.

## Results

Clinical characteristics are presented in Table 1. Male gender was most prevalent in MCL (77.2%) and least prevalent in MZL (47.4%), and the percent of cases older than 60 years was the highest for MCL (63.7%) and lowest for HL (20.9%) as expected (Table 1). At a median follow-up of 5.9 years (range, 0.02–14.1 years), 1071 participants (44.6%) had an event and 613 participants (25.6%) had died.

**Table 1:** Demographic and clinical characteristics

Subtype	Covariate	Category	*N*	%
DLBCL *N* = 736	Sex	Male	413	56.1%
	Age, years	>60	432	58.7%
	IPI	0 - Low risk	88	12.0%
		1 - Low risk	174	23.6%
		2 - Low-intermediate risk	226	30.7%
		3 - High-intermediate risk	177	24.0%
		4 - High risk	58	7.9%
		5 - High risk	13	1.8%
	PS	<2	659	89.5%
		≥2	77	10.5%
	Treatment	Immunochemotherapy	668	90.8%
FL *N* = 703	Sex	Male	361	51.4%
	Age, years	>60	360	51.2%
	FLIPI	0 - Low risk	78	11.1%
		1 - Low risk	211	30.0%
		2 - Intermediate risk	240	34.1%
		3 - High risk	130	18.5%
		4 - High risk	37	5.3%
		5 - High risk	7	1.0%
	PS	<2	687	97.7%
		≥2	16	2.3%
	FLIII	No	601	85.5%
		Yes	102	14.5%
	Treatment	Observation	249	35.4%
		R monotherapy	89	12.7%
		Immunochemotherapy	272	38.7%
		Other chemotherapy	93	13.2%
MZL *N* = 302	Sex	Male	143	47.4%
	Age, years	>60	166	55.0%
	IPI	0 - Low risk	75	24.8%
		1 - Low risk	121	40.1%
		2 - Low-intermediate risk	71	23.5%
		3 - High-intermediate risk	32	10.6%
		4 - High risk	3	1.0%
		5 - High risk	0	0.0%
	PS	<2	298	98.7%
		≥2	4	1.3%
MCL *N* = 193	Sex	Male	149	77.2%
	Age, years	>60	123	63.7%
	MIPI	Low risk (0–3)	80	41.5%
		Interm/high risk (4–12)	113	58.5%
	PS	<2	181	93.8%
		≥2	12	6.2%
HL N = 297	Sex	Male	155	52.2%
	Age, years	>60	62	20.9%
	IPS	0 - Low risk	1	.3%
		1 - Low risk	47	15.8%
		2 - Low risk	116	39.1%
		3 - Intermediate risk	81	27.3%
		4 - Intermediate risk	35	11.8%
		5 - High risk	12	4.0%
		6 - High risk	5	1.7%
	PS	< 2	280	94.3%
		≥ 2	17	5.7%
TCL *N* = 168	Sex	Male	104	61.9%
	Age, years	> 60	77	45.8%
	IPI	0 - Low risk	33	19.6%
		1 - Low risk	45	26.8%
		2 - Low-intermediate risk	45	26.8%
		3 - High-intermediate risk	29	17.3%
		4 - High risk	15	8.9%
		5 - High risk	1	.6%
	PS	< 2	146	86.9%
		≥ 2	22	13.1%

Abbreviations: *DLBCL* diffuse large B-cell lymphoma, *FL* follicular lymphoma, *FLIPI* Follicular Lymphoma International Prognostic Index, *FLIII* follicular lymphoma grade 3*, HL* Hodgkin lymphoma, *IPI* International Prognostic Index, *MCL* mantle cell lymphoma, *MIPI* Mantle Cell International Prognostic Index, *MZL* marginal zone lymphoma, *PS* performance status, *TCL* T-cell lymphoma

**Fig. 1:**
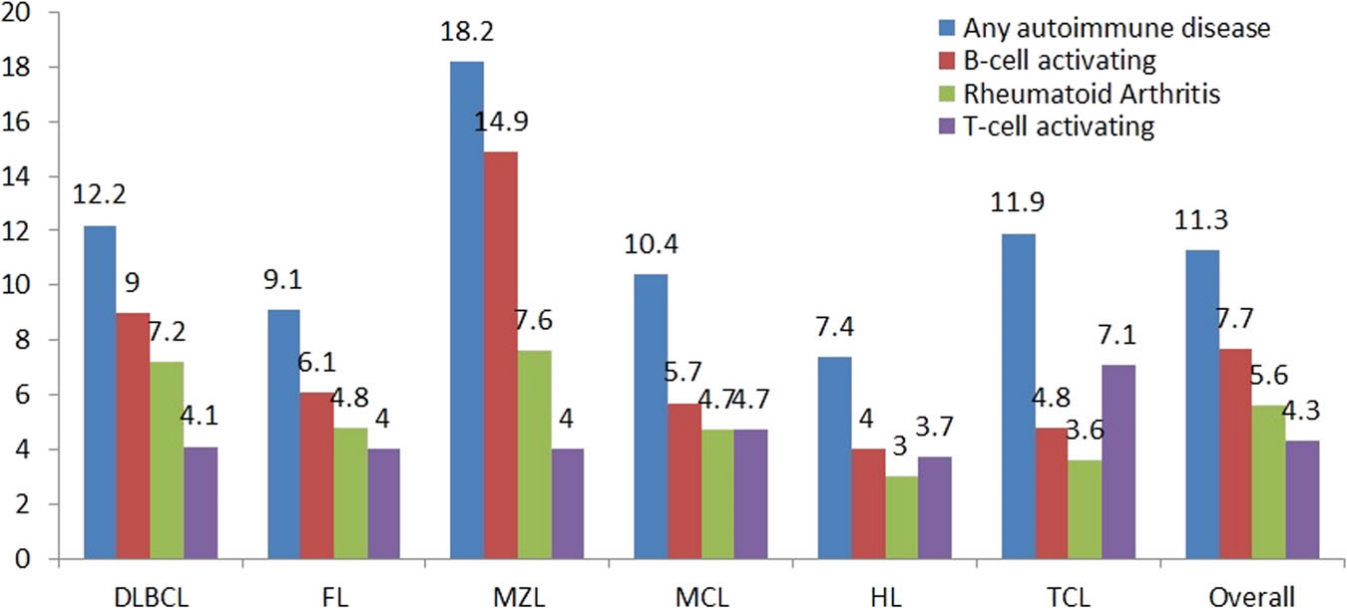
**Prevalence of autoimmune conditions, B-cell/T-cell-activating autoimmune conditions, and rheumatoid arthritis by lymphoma subtypes**. Abbreviations: *DLBCL* diffuse large B-cell lymphoma, *FL* follicular lymphoma, *HL* Hodgkin lymphoma, *MCL* mantle cell lymphoma, *MZL* marginal zone lymphoma, *TCL* T-cell lymphoma

The prevalence of any of the eight self-reported autoimmune conditions varied across subtypes and was highest in MZL (18.2%), followed by DLBCL (12.2%), TCL (11.9%), MCL (10.4%), FL (9.1%), and HL (7.4%). Autoimmune conditions primarily mediated by B-cell responses were more prevalent than autoimmune conditions primarily mediated by T-cell responses in DLBCL (9.0% vs. 4.1%), FL (6.1% vs. 4.0%), MZL (14.9% vs. 4.0%), and MCL (5.7% vs. 4.7%), similar in HL (4.0% vs. 3.7%), whereas autoimmune conditions primarily mediated by T-cell responses were more prevalent than autoimmune conditions primarily mediated by B-cell responses in TCL (7.1% vs. 4.8%). RA was the most common autoimmune condition and was highest in MZL (7.6%), followed by DLBCL (7.2%), FL (4.8%), MCL (4.7%), TCL (3.6%), and HL (3.0%) (Fig. 1).

History of an autoimmune condition was associated with female gender (58.7% with any autoimmune condition vs. 43.0% without autoimmune condition, *P* < 0.001), age > 60 years (60.1% with any autoimmune condition vs. 49.7% without autoimmune condition, *P* = 0.001), smoking status (*P* < 0.001; mainly driven by missing status), and lymphoma subtype (*P* < 0.001; strongest for MZL), but was not associated with ECOG performance status ≥ 2 (8.1% with any autoimmune condition vs. 5.9% without autoimmune condition, *P* = 0.16), high prognostic index (10.3% with any autoimmune condition vs. 11.9% without autoimmune condition, *P* = 0.44), or the presence of comorbidities (Table 2).

**Table 2:** Clinical characteristics by any autoimmune conditions*

		No autoimmune condition		Any autoimmune condition		
Characteristic		*N*	%	*N*	%	*P*
Sex	Male	1213	57.0%	112	41.3%	< 0.001
	Female	915	43.0%	159	58.7%	
Age	≤ 60 Years	1071	50.3%	108	39.9%	0.001
	> 60 Years	1057	49.7%	163	60.1%	
Performance Status	< 2	2002	94.1%	249	91.9%	0.16
	≥ 2	126	5.9%	22	8.1%	
Prognostic Index	Low-intermediate risk	1874	88.1%	243	89.7%	0.44
	High risk	254	11.9%	28	10.3%	
Comorbidities	No	1543	72.5%	179	66.1%	0.08
	Yes	237	11.1%	38	14.0%	
	Missing	348	16.4%	54	19.9%	
Smoking	Never	971	45.6%	119	43.9%	< 0.001
	Former	542	25.5%	94	34.7%	
	Current	160	7.5%	26	9.6%	
	Missing	455	21.4%	32	11.8%	
Subtype	DLBCL	646	30.4%	90	33.2%	< 0.001
	FL	639	30.0%	64	23.6%	
	MZL	247	11.6%	55	20.3%	
	MCL	173	8.1%	20	7.4%	
	HL	275	12.9%	22	8.1%	
	TCL	148	7.0%	20	7.4%	

*Based on self-report. *DLBCL* diffuse large B-cell lymphoma, *HL* Hodgkin lymphoma, *IPI* International Prognostic Index, *MALT* mucosa-associated lymphoid tissue, *MCL* mantle cell lymphoma, *MZL* marginal zone lymphoma, *PS* performance status, *TCL* T-cell lymphoma

**Fig. 2:**
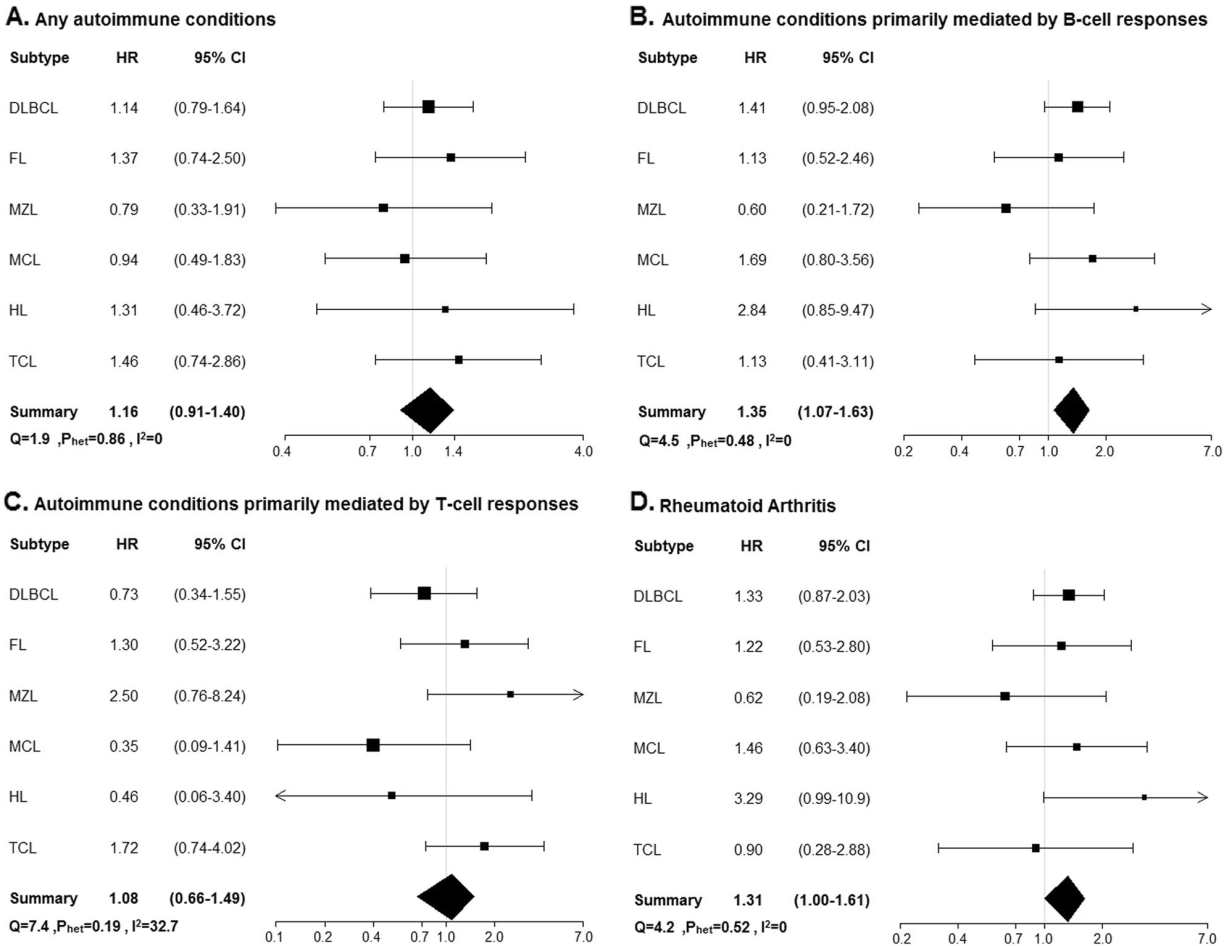
**Overall survival for any autoimmune conditions, B-cell/T-cell-activating autoimmune conditions, and rheumatoid arthritis**. Abbreviations: *CI* confidence interval, *DLBCL* diffuse large B-cell lymphoma, *FL* follicular lymphoma, *MZL* marginal zone lymphoma, *HL* Hodgkin lymphoma, *HR* hazard ratio, *I*^2^ statistic to quantify the proportion of the total variation due to heterogeneity, *MCL* mantle cell lymphoma, *P*_het_ test for heterogeneity across lymphoma subtypes, *Q* Cochran’s *Q*-statistic, *TCL* T-cell lymphoma. **a** Association between overall survival and any autoimmune disease across lymphoma subtypes. **b** Association between overall survival and autoimmune conditions primarily mediated by B-cell responses across lymphoma subtypes. **c** Association between overall survival and autoimmune conditions primarily mediated by T-cell responses across lymphoma subtypes. **d** Association between overall survival and rheumatoid arthritis across lymphoma subtypes

**Fig. 3:**
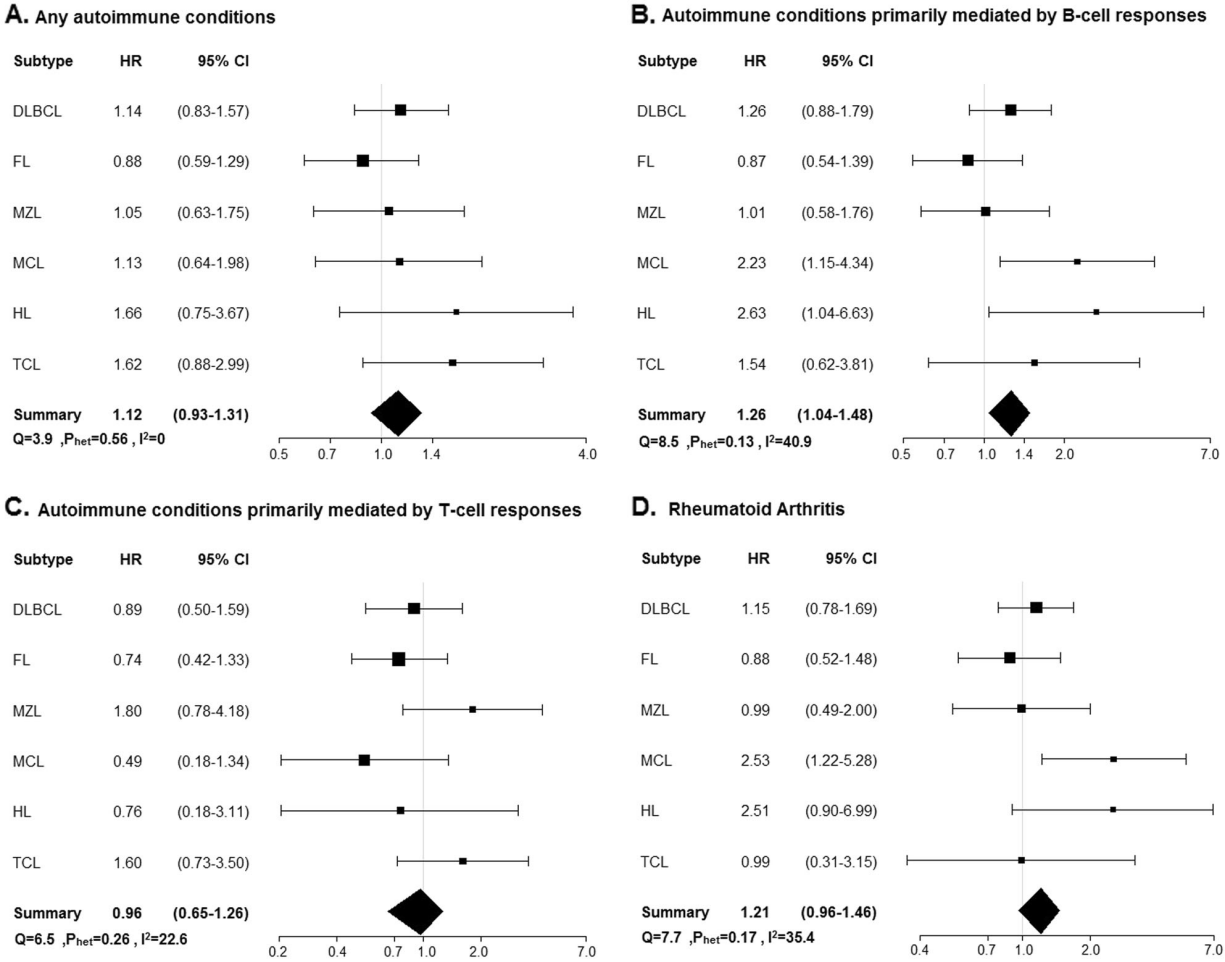
**Event-free survival for any autoimmune conditions, B-cell/T-cell activating autoimmune conditions, and rheumatoid arthritis**. Abbreviations: *CI* confidence interval, *DLBCL* diffuse large B-cell lymphoma, *FL* follicular lymphoma, *HL* Hodgkin lymphoma, *HR* hazard ratio, *I*^*2*^ statistic to quantify the proportion of the total variation due to heterogeneity, *MCL* mantle cell lymphoma, *MZL* marginal zone lymphoma, *P*_het_ test for heterogeneity across lymphoma subtypes, *Q* Cochran’s *Q*-statistic, *TCL* T-cell lymphoma. **a** Association between event-free survival and any autoimmune disease across lymphoma subtypes. **b** Association between event free survival and autoimmune conditions primarily mediated by B-cell responses across lymphoma subtypes. **c** Association between event-free survival and autoimmune conditions primarily mediated by T-cell responses across lymphoma subtypes. **d** Association between event free survival and rheumatoid arthritis across lymphoma subtypes

There was no evidence of an association of history of any autoimmune condition as a group with EFS or OS for any lymphoma subtype (Table 3 and Figs. 2, 3). However, there were positive associations of autoimmune conditions primarily mediated by B-cell responses with inferior EFS in MCL (HR = 2.23, 95% CI 1.15–4.34) and HL (HR = 2.63, 95% CI 1.04–6.63), which was largely driven by RA, the most common autoimmune condition. Similar associations with OS were seen in MCL (HR = 1.69, 95% CI 0.80–3.56) and HL (HR = 2.84, 95% CI 0.85–9.47), but these were not statistically significant; there was also a trend toward inferior OS for DLBCL (HR = 1.41, 95% CI 0.95–2.08).

**Table 3:** Multivariable-adjusted hazard ratios for any autoimmune conditions, B-cell/T-cell-activating autoimmune conditions, and rheumatoid arthritis by lymphoma subtype

			Prevalence of autoimmune condition		Event-free survival					Overall survival				
					Events					Events				
		Adjustment factors	*N*	%	*N*	%	HR	95% CI	*p*	*N*	%	HR	95% CI	*p*
DLBCL (*N* = 736)	No autoimmune condition	Sex, IPI, treatment*	646		286	44.3	1.00	Reference		215	33.3	1.00	Reference	
	Any autoimmune condition		90	12.2	44	48.9	1.14	0.83–1.57	0.42	33	36.7	1.14	0.79–1.64	0.49
	B-cell responses		66	9.0	35	53.0	1.26	0.88–1.79	0.20	29	43.9	1.41	0.95–2.08	0.09
	Rheumatoid arthritis		53	7.2	28	52.8	1.15	0.78–1.69	0.49	24	45.3	1.33	0.87–2.03	0.18
	T-cell reponses		30	4.1	12	40.0	0.89	0.50–1.59	0.70	7	23.3	0.73	0.34–1.55	0.41
FL (*N* = 703)	No autoimmune disease	Sex, FLIPI, FLIII, treatment^	639		332	52.0	1.00	Reference		110	17.2	1.00	Reference	
	Any autoimmune condition		64	9.1	29	45.3	0.88	0.59–1.29	0.50	12	18.8	1.37	0.74–2.50	0.32
	B-cell activating		43	6.1	19	44.2	0.87	0.54–1.39	0.55	7	16.3	1.13	0.52–2.46	0.76
	Rheumatoid arthritis		34	4.8	15	44.1	0.88	0.52–1.48	0.62	6	17.6	1.22	0.53–2.80	0.64
	T-cell activating		28	4.0	12	42.9	0.74	0.42–1.33	0.32	5	17.9	1.30	0.52–3.22	0.58
MZL (*N* = 302)	No autoimmune condition	Sex, IPI	247		88	35.6	1.00	Reference		35	14.2	1.00	Reference	
	Any autoimmune condition		55	18.2	19	34.5	1.05	0.63–1.75	0.85	6	10.9	0.79	0.33–1.91	0.79
	B-cell activating		45	14.9	15	33.3	1.01	0.58–1.76	0.98	4	8.9	0.60	0.21–1.72	0.34
	Rheumatoid arthritis		23	7.6	9	39.1	0.99	0.49–2.00	0.98	3	13.0	0.62	0.19–2.08	0.44
	T-cell activating		12	4.0	6	50.0	1.80	0.78–4.18	0.17	3	25.0	2.50	0.76–8.24	0.13
MALT (*N* = 219)	No autoimmune condition	Sex, IPI	173		59	34.1	1.00	Reference		18	10.4	1.00	Reference	
	Any autoimmune condition		46	21	16	34.8	1.38	0.77–2.49	0.29	3	6.5	1.08	0.30–3.80	0.91
	B-cell activating		39	17.8	13	33.3	1.33	0.71–2.49	0.37	2	5.1	0.86	0.19–3.82	0.84
	Rheumatoid arthritis		18	8.2	7	38.9	1.39	0.64–3.04	0.41	1	5.6	0.73	0.10–5.52	0.76
	T-cell activating		8	3.7	4	50.0	1.80	0.64–5.07	0.26	1	12.5	1.83	0.24–14.1	0.56
MCL (*N* = 193)	No autoimmune condition	Sex, MIPI	173		105	60.7	1.00	Reference		77	44.5	1.00	Reference	
	Any autoimmune condition		20	10.4	14	70.0	1.13	0.64–1.98	0.68	10	50.0	0.94	0.49–1.83	0.94
	B-cell activating		11	5.7	10	90.9	**2.23**	**1.15–4.34**	**0.02**	8	72.7	1.69	0.80–3.56	0.17
	Rheumatoid arthritis		9	4.7	8	88.9	**2.53**	**1.22–5.28**	**0.01**	6	66.7	1.46	0.63–3.40	0.38
	T-cell activating		9	4.7	4	44.4	0.49	0.18–1.34	0.17	2	22.2	0.35	0.09–1.41	0.35
HL (*N* = 297)	No autoimmune condition	Sex, IPS	275		53	19.3	1.00	Reference		33	12.0	1.00	Reference	
	Any autoimmune condition		22	7.4	7	31.8	1.66	0.75–3.67	0.21	4	18.2	1.31	0.46–3.72	0.61
	B-cell activating		12	4.0	5	41.7	**2.63**	**1.04–6.63**	**0.04**	3	25.0	2.84	0.85–9.47	0.09
	Rheumatoid arthritis		9	3.0	4	44.4	2.51	0.90–6.99	0.08	3	33.3	3.29	0.99–10.9	0.05
	T-cell activating		11	3.7	2	18.2	0.76	0.18–3.11	0.7	1	9.1	0.46	0.06–3.40	0.45
TCL (*N* = 168)	No autoimmune condition	Sex, IPI	148		82	55.4	1.00	Reference		68	45.9	1.00	Reference	
	Any autoimmune condition		20	11.9	12	60.0	1.62	0.88–2.99	0.12	10	50.0	1.46	0.74–2.86	0.28
	B-cell activating		8	4.8	5	62.5	1.54	0.62–3.81	0.35	4	50.0	1.13	0.41–3.11	0.81
	Rheumatoid arthritis		6	3.6	3	50.0	0.99	0.31–3.15	0.99	3	50.0	0.90	0.28–2.88	0.86
	T-cell activating		12	7.1	7	58.3	1.60	0.73–3.50	0.24	6	50.0	1.72	0.74–4.02	0.21

Abbreviations: *CI* confidence interval, *DLBCL* diffuse large B-cell lymphoma, *FL* follicular lymphoma, *FLIPI* Follicular Lymphoma International Prognostic Index, *FLIII* follicular lymphoma grade 3, *HL* Hodgkin lymphoma, *HR* hazard ratio, *IPI* International Prognostic Index, *IPS* International Prognostic Score, *MALT* mucosa-associated lymphoid tissue, *MCL* mantle cell lymphoma, *MIPI*, Mantle Cell International Prognostic Index, *MZL* marginal zone lymphoma, *TCL* T-cell lymphoma

*Immunochemotherapy vs. all other therapy. ^Rituximab-based therapy, other chemotherapy vs. observation.

Bold would be considered statistically significant

To further address potential confounding by comorbidity or smoking status, we re-ran models in Table 3 that included both of these variables. After these further adjustments, we observed slightly stronger associations of autoimmune conditions primarily mediated by B-cell responses with inferior EFS in MCL (HR = 2.36, 95% CI 1.17–4.75) and HL (HR = 2.73, 95% CI 1.06–7.04), which was largely driven by RA for both MCL (HR = 3.08, 95% CI 1.39–6.82) and HL (HR = 2.73, 95% CI 0.92–7.47), whereas similar associations were observed with OS for both MCL (HR = 1.71, 95% CI 0.77–3.77) and HL (HR = 3.05, 95% CI 0.88–10.6). Results for all other subtypes in Table 3 were not changed by these adjustments (data not shown).

Autoimmune conditions primarily mediated by T-cell responses were not associated with EFS or OS for any subtype, although the number of events was generally small for this exposure.

## Discussion

Although we found no overall association of any autoimmune disease as a group with prognosis for individual lymphoma subtypes, we did find that a history of autoimmune conditions primarily mediated by B-cell responses were associated with inferior EFS for MCL and HL, and that this was largely driven by RA. The latter associations showed similar but not statistically significant trends with OS. Although autoimmune conditions primary mediated by T-cell responses were not associated with any of the lymphoma subtypes, these analyses were challenged by the low prevalence of these types of autoimmune diseases.

Strengths of this study include the following: the prospective study design; central pathology review; use of the InterLymph autoimmune classification^27^; availability of high-quality clinical and outcome data, including both EFS and OS; and adjustment for subtype-specific prognostic factors, as well as comorbidity and smoking status. Limitations of our study include the following: self-reported data for autoimmune conditions; ascertainment of only eight types of autoimmune conditions, whereas there are ~80 types of autoimmune diseases; and likely limited power to assess some associations, particularly for autoimmune conditions primarily mediated by T-cell responses and for OS. Self-reported autoimmune conditions can lead to exposure misclassification, although a case–control study from InterLymph that used self-reported data for autoimmune conditions found a good concordance between control prevalence with population prevalence from different countries, except for RA and ulcerative colitis, which were higher than published prevalence estimates^17^. The prevalence of RA in our DLBCL cases (7.2%) was also higher than the prevalence reported by study using Surveillance, Epidemiology, and End Results (SEER)-Medicare data (2.6%)^23^, but it is not clear if this is a function of differences in study design, study population characteristics, or over-reporting of these conditions in our study. Our study was also not able to capture measures of severity of autoimmune conditions, treatment for autoimmune conditions, adjustments to lymphoma-directed therapy based on autoimmune disease status (including curative intent in aggressive lymphomas), nor use of more recent immune therapies such as CAR-T and immune checkpoint inhibitors, all of which may have an impact on lymphoma prognosis and are important unaddressed needs for future research.

Our findings are supported in part by other studies, although findings from the small number of studies published to date are not consistent. A population-based cohort study of 1523 Swedish NHL patients with a median follow-up of 8.8 years, found a 1.4-fold increased risk of death among NHL cases with autoimmune conditions compared with those without autoimmune conditions, which included both B-cell- and T-cell-mediated autoimmune conditions (RA, SS, SLE, and celiac disease); however, they did not find an association with subtype-specific lymphomas including DLBCL, FL, MCL, or TCL^18^. An Israeli study of 435 B-cell NHL cases with a median follow-up of 3.5 years found a 1.69-fold increased risk of relapse among cases with any autoimmune conditions, which included both B-cell- and T-cell-mediated autoimmune conditions, and a 3.41-fold increased risk of relapse among cases with autoimmune conditions primarily mediated by B-cell responses when compared with patients without autoimmune conditions^24^. Another Swedish study examined the impact of concomitant RA on cause-specific survival and OS among 329 NHL and 60 HL patients, and reported a 1.43-fold increased risk for cause-specific survival among RA cases with NHL, and a 1.41-fold and 1.33-fold increased risk of death among RA cases with NHL and HL, respectively^19^. Our study differed from the Swedish study by collecting autoimmune status by patient self-report as opposed to registry data.

Conversely, a study using the Nebraska Lymphoma Study Group and the Mayo Clinic Lymphoma Database registries of 1595 NHL patients found that RA was associated with improved NHL-related outcomes, with a 40% reduced risk of death and a 60% lower risk of lymphoma relapse or progression compared with non-RA NHL patients; however, patients with concomitant RA and NHL were more than twice as likely to die from causes unrelated to lymphoma^20^. A study in Taiwan retrospectively reviewed 913 medical records of newly diagnosed lymphoma patients and found that pre-existing autoimmune diseases, which included B-cell- and T-cell-mediated autoimmune diseases, and other thyroid autoimmune diseases, were not associated with inferior progression-free survival or OS^22^. A study using the SEER database with 5926 DLBCL patients, examined survival patterns in DLBCL cases with RA, SLE, SS, and other B-cell-mediated autoimmune diseases, and found no significant difference compared to patients with no history of these diseases^23^. In addition, a study from the University of Iowa/Mayo Clinic Molecular Epidemiology Resource found that a history of immunosuppression did not affect subsequent prognosis in DLBCL cases, although it is known to be a risk factor for the development of DLBCL.^32^ The latter finding aligns with our DLBCL survival findings and the Swedish findings^18^, although we did see a trend towards inferior survival in DLBCL patients with autoimmune conditions primarily mediated by B-cell responses.

In contrast, in the Israeli study, autoimmune conditions primarily mediated by B-cell responses compared to those without autoimmune conditions primarily mediated by B-cell responses were associated with an increased risk of relapse (HR = 3.83; 95% CI: 1.20–12.3) and death (HR = 8.34; 95% CI: 3.01–23.1) in 182 patients with DLBCL and an increased risk of relapse (HR = 13.4; 95% CI: 2.48–72.6) in 65 patients with MZL.^24^ However, autoimmune conditions were relatively rare in this study, which explains the imprecise CIs. In addition, there was some evidence in our study of a positive trend between autoimmune conditions primarily mediated by T-cell responses and EFS and OS for TCL, although it was not statistically significant.

Integrating these findings with the etiology findings from the InterLymph pooled case–control analyses, which found that specific NHL subtypes are associated with distinct autoimmune diseases, supports the hypothesis that there are likely to be subtype-specific mechanisms of lymphomagenesis, mechanisms that have yet to be elucidated but which may provide new biologic insights^10–12,17^. For example, our data suggest that there may be a mechanism involving lymphomas originating from B-lymphocytes, specifically MCL, HL, and perhaps DLBCL, and autoimmune conditions primarily mediated by B-cell responses, and lymphomas originating from T-lymphocytes, specifically TCL, and autoimmune conditions primarily mediated by T-cell responses in terms of lymphoma pathogenesis and prognosis.

Alternately, it has been proposed that the level of inflammation and severity of the autoimmune condition may contribute to increased risk of lymphoma development. Major predisposing factors for lymphoma development include chronic activation or stimulation of B-cells or T-cells, and the type of autoimmune condition involved in lymphoma pathogenesis is likely disease-specific^33^. For example, aggressive systemic inflammation in RA cases increases chronic activation of peripheral B-cells that in turn may increase clonal B-cell populations, which may lead to DLBCL^33,34^. In celiac disease, proliferation of T-cells at the inflammation site may predispose for enteropathy-associated TCL^33^. However, less is known about how these possible biological mechanisms may contribute to prognosis once lymphoma developed in patients with autoimmune conditions, which also must incorporate disease activity and ongoing treatment(s) for both the autoimmune disease and the lymphoma, and these are important areas for future research, particularly with new immune-based therapies for the treatment of lymphomas.

Further validation is needed for these findings, and larger sample sizes, such as large institutional studies with high-quality data on autoimmune conditions, including severity and treatment, linked with lymphoma outcomes appear to be warranted to fully understand the role of these diseases in the management of lymphoma patients. Specifically, studies are warranted to replicate our findings for MCL and HL, and perhaps a larger study for TCL that will have enough power to investigate the association of prognosis with autoimmune conditions primarily mediated by T-cell responses.
